# Ertapenem susceptibility of extended spectrum *beta*-lactamase-producing organisms

**DOI:** 10.1186/1476-0711-6-6

**Published:** 2007-06-06

**Authors:** Rupal M Mody, Daniel P Erwin, Amy M Summers, Hector A Carrero, Edward B Selby, Allesa J Ewell, Kimberly A Moran

**Affiliations:** 1 Walter Reed Army Medical Center, Washington, DC 20307, USA; 2 Uniformed Services University of the Health Sciences, Bethesda, MD 20814, USA

## Abstract

**Background:**

Infections caused by multiply drug resistant organisms such as extended spectrum *beta*-lactamase (ESBL)-producing *Escherichia coli *and *Klebsiella pneumoniae *are increasing. Carbapenems (imipenem and meropenem) are the antibiotics commonly used to treat these agents. There is limited clinical data regarding the efficacy of the newest carbapenem, ertapenem, against these organisms. Ertapenem susceptibility of ESBL-producing *E. coli *and *K. pneumoniae *clinical isolates were evaluated and compared to imipenem to determine if imipenem susceptibility could be used as a surrogate for ertapenem susceptibility.

**Methods:**

100 ESBL isolates (n = 34 *E. coli *and n = 66 *K. pneumoniae*) collected from 2005–2006 clinical specimens at WRAMC were identified and tested for susceptibility by Vitek Legacy [bioMerieux, Durham, NC]. Ertapenem susceptibility was performed via epsilometer test (E-test) [AB Biodisk, Solna, Sweden].

**Results:**

100% of ESBL isolates tested were susceptible to ertapenem. 100% of the same isolates were also susceptible to imipenem.

**Conclusion:**

These results, based on 100% susceptibility, suggest that ertapenem may be an alternative to other carbapenems for the treatment of infections caused by ESBL-producing *E. coli *and *K. pneumoniae*. Clinical outcomes studies are needed to determine if ertapenem is effective for the treatment of infection caused by these organisms. However, due to lack of resistant isolates, we are unable to conclude whether imipenem susceptibility accurately predicts ertapenem susceptibility.

## Background

Military operations in Southwest Asia have led to an increase in patients at Walter Reed Army Medical Center (WRAMC) admitted with traumatic injury and complicated infections caused by multiply drug resistant organisms such as extended spectrum *beta*-lactamase (ESBL)-producing *Escherichia coli *and *Klebsiella pneumoniae *[1]. Outpatient antibiotic regimens for the war wounded have become limited due to the extreme resistance of these organisms. Mortality from infections caused by these organisms may range from 42–100% if not treated with appropriate antibiotics [2]. **Carbapenems **[imipenem (IPM) and meropenem (MEM)] are the drugs of choice to treat infection caused by ESBL-producing organisms [2-5]. However, the multiple daily dosing required for these antibiotics make them an onerous treatment regimen for soldiers once they have left the hospital.

There is limited clinical data regarding ertapenem (ETP), a relatively new carbapenem, against ESBL-producing organisms [6,7]. However, the ease of once daily dosing and reduced cost of ETP at $27.67/day vs. $60–$71/day for IPM and MEM make this drug a desired alternative in the treatment of the war wounded, especially those requiring a prolonged treatment course (Amerisource Bergen, Cardinal Health, Federal Supply Schedule drug prices). The aim of this project is to evaluate ETP susceptibility of ESBL-producing *E. coli *and *K. pneumoniae *clinical isolates. A secondary aim is to compare IPM and ETP susceptibilities to determine if IPM can be used as a surrogate for ETP susceptibility.

## Methods

All isolates are culled from prior 2005–2006 clinical specimens. Organism identification, screening for ESBL production, and antimicrobial susceptibility testing are performed using Vitek Legacy (bioMerieux, Durham, NC). Isolates flagged by Vitek as possible ESBL producers are submitted for disk diffusion confirmatory testing. Phenotypic confirmatory disk diffusion testing is done on presumptive ESBL-producing organisms in accordance with the Clinical and Laboratory Standards Institute [8].

ETP susceptibility is determined using the epsilometer (E-test) [AB Biodisk, Solna, Sweden] method as per manufacturer's protocol [9]. Briefly, isolates are revived from freezer stocks by passing twice to 5% Sheep Blood Agar plates (Remel, Lenexa, KS). Select isolated colonies produce a 0.5 McFarland suspension in sterile saline. This suspension is inoculated onto Mueller-Hinton agar plates which the antibiotic E-test strip was placed. Each ETP E-test strip consists of a predefined gradient of antibiotic allowing Minimum Inhibitory Concentration (MIC) measurements through the range of 0.002–32 ug/mL. MIC's for each isolate are determined by reading the value at the point of intersection between the zone of inhibition edge and the E-test strip.

**Figure 1:**
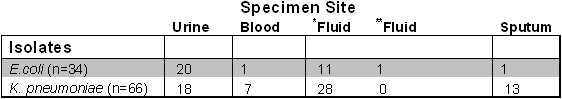
Specimen location for ESBL isolates. The symbol (*) includes wounds and sterile body fluids and the symbol (**) includes fluid from a JP drain.

Susceptibility data to ETP is determined as per the Clinical and Laboratory Standards Institute interpretive criteria (4). For ETP, sensitive is defined as MIC</= 2 ug/mL, intermediate 4 ug/mL, and resistant >/= 8 ug/mL. Data compiled for 100 clinical isolates (n = 34 *E. coli *and n = 66 *K. pneumoniae*) includes specimen site for each isolate (Figure 1).

## Results

100% of ESBL-producing clinical isolates show susceptibility to ETP as per CLSI criteria. ETP MICs range from 0.006 ug/mL to 0.5 ug/mL (mean 0.073, SD 0.093) [95% CI 0.032 ug/mL] for *E. coli*. and 0.006 ug/mL to 2 ug/mL (mean 0.125, SD 0.291) [95% CI 0.070] for *K. pneumoniae*.

**Figure 2:**
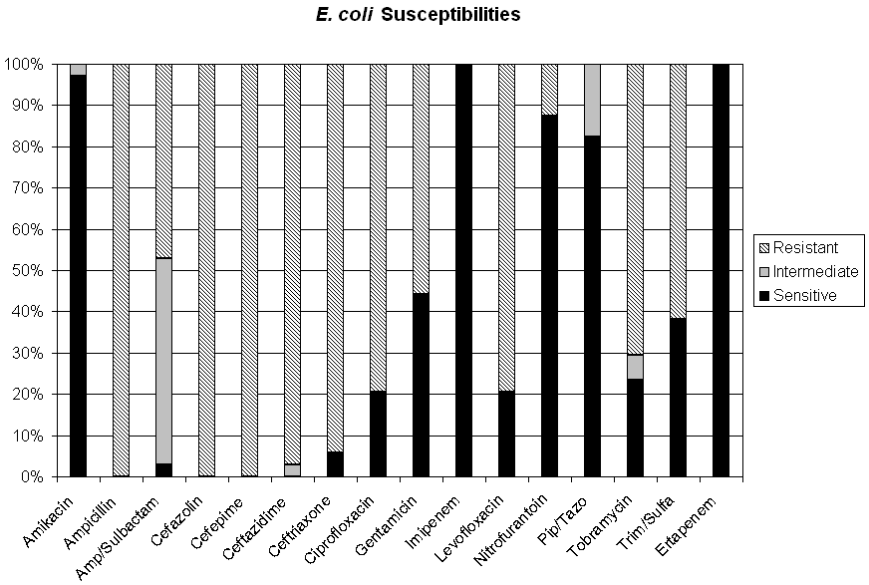
ESBL *E.coli *susceptibilities to ertapenem and other antibiotics. Solid black represents percentage of isolates sensitive to antibiotics. Light gray represents percentage of isolates intermediately sensitive to antibiotics. Diagonal lines represent percentage of isolates resistant to antibiotics.

**Figure 3:**
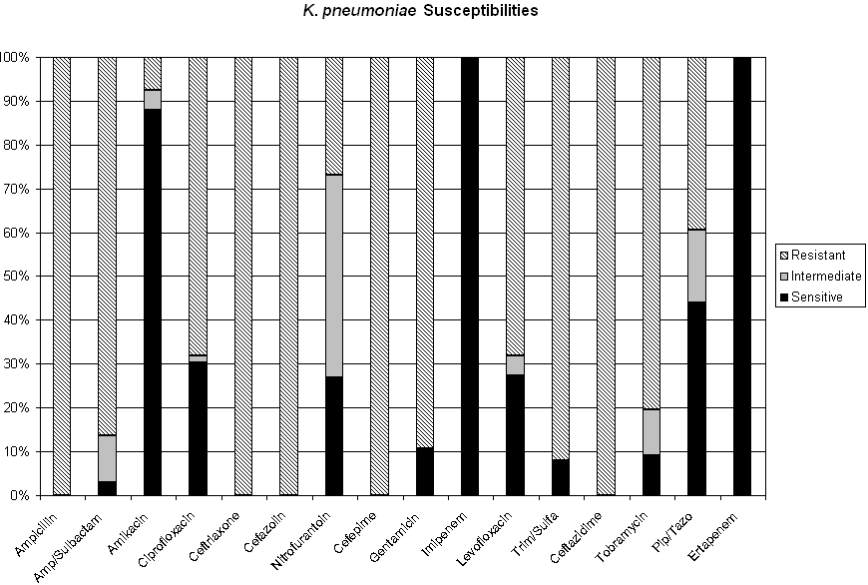
ESBL K. pneumoniae susceptibilities to ertapenem and other antibiotics. Solid black represents percentage of isolates sensitive to antibiotics. Light gray represents percentage of isolates intermediately sensitive to antibiotics. Diagonal lines represent percentage of isolates resistant to antibiotics.

100% of isolates susceptible to ETP are also susceptible to IPM by Vitek (MIC </= 4). In regards to susceptibility of ESBL-producing organisms to other antibiotics, 21% of ESBL *E. coli *and 27% of ESBL *K. pneumoniae *are susceptible to a quinolone such as levofloxacin. ESBL *E. coli *and *K. pneumoniae *are 97% and 88% sensitive to amikacin (aminoglycoside) respectively. Cephalosporin sensitivity shows approximately 100% resistance to cefepime and ceftazidime for all clinical specimens (Figures 2 and 3).

## Discussion

100% of the clinical isolates of ESBL-producing *E. coli *and *K. pneumoniae *tested were susceptible to ertapenem in this study with MICs ranging from 0.006 to 0.5 ug/mL for *E. coli *and 0.006 to 2 ug/mL for *K. pneumoniae*. Prior studies showed similar ETP efficacy for ESBL-producing organisms *in vitro *[6,7].

Various studies report *in vivo *ETP susceptibility of clinical isolates. Livermore et al. tested 181 ESBL-producing clinical Enterobacteriaceae isolates (all *Klebsiella *spp.) taken from ICU patients and found ertapenem to inhibit 90% of isolates but was found to be less active against ESBL nonproducers [7]. Betriu et al. tested 70 clinical ESBL-producing Enterobacteriaceae isolates which were found to be 98.6% susceptible to ertapenem at 10^4 ^and 10^6 ^inoculum with MICs ranging from 0.03–0.12 mg/L [6]. In our study, inoculum size is held constant and plays no role in evaluation of ETP susceptibility, so no inferences can be made based on inoculum size.

Susceptibility of ESBL-producing isolates in this study differed in regards to aminoglycoside and quinolone sensitivity. Overall, greater than 80% of the clinical isolates show sensitivity to amikacin, while less than 30% are sensitive to levofloxacin suggesting that aminoglycosides may be appropriate empiric adjunct therapy if concern over ESBL-producing organisms exist in the critically ill patient. Our data in conjunction with other studies suggest that ETP may be an alternative to other carbapenems in the treatment of infections caused by ESBL-producing organisms. Ertapenem use to treat these infections would offer the benefit of ease of administration with once daily dosing (t_1/2 _4–4.5 hrs) and reduced cost. These advantages to the use of ETP are of particular value to clinicians and patients at Walter Reed Army Medical Center due to the role of ESBL-producing organisms in infections such as osteomyelitis which require a prolonged course of antibiotics. This is of importance because current use of meropenem and imipenem to treat these infections with their burdensome multiple daily dosing regimens often results in anger and frustration in our outpatient soldiers.

All isolates tested are susceptible to ertapenem and imipenem, which has also been observed in prior studies [6,7]. However, due to lack of resistant isolates, we are unable to confidently conclude whether IPM susceptibility could accurately predict ETP susceptibility.

Although no resistance is shown in this study, ETP use should be exercised with caution in light of reports of recent ETP resistance both *in vivo *and *in vitro *to ESBL-producing *E. coli *and *K. pneumoniae *[10-13]. The maximum ETP MIC of 2 ug/ml was seen in a *Klebsiella pneumoniae *clinical isolate with corresponding MIC to IPM <4 ug/ml. It has been noted in multiple studies that increasing MIC of ETP typically corresponds with increased MIC of IPM/MEM (especially MIC 4–8, intermediate susceptibility range for IPM/MEM). In a report by Lartigue, ETP resistance (MIC>256 mg/L) shown in ESBL *E.coli *was associated with intermediate resistance (MIC 8 mg/L for IPM/MEM) emphasizing the greater likelihood of resistance developing to ETP than to other carbapenems [12]. Paterson et al. report *in vivo *resistance to ESBL *K. pneumoniae *in which 10.9% of ESBL *K. pneumoniae *isolates collected from intra-abdominal sources worldwide were resistant to ETP [13]. After producing organisms deficient in outer membrane proteins or porins, Omp 35 and Omp 36, Jacoby et al. demonstrated *in vitro *resistance of ESBL *K. pneumoniae *to ETP [11]. A similar mechanism of *in vivo *resistance involving lack of outer membrane proteins has recently been reported by Lartigue et al. for ESBL *E. coli *from a peritoneal fluid culture [12].

Weaknesses of this study include unknown inoculum effect, lack of non-ESBL-producing controls, and theoretically, interobserver variability in interpretation of ETP E-test sensitivity data. Strengths include the large number of diverse isolates culled from various clinical sources for susceptibility testing.

It is difficult to extrapolate *in vitro *data to clinical practice. Although *in vitro *studies show ETP is promising in the treatment of ESBL organisms, further clinical studies are necessary. However, ETP should be kept in mind when seeking a safe, effective, easy outpatient antibiotic regimen for the treatment of the complicated infections in our war wounded.

## Conclusion

The results of our data show that ESBL *Klebsiella pneumoniae *and ESBL *Escherichia coli *isolates clinical isolates culled from soldiers at WRAMC showed uniform ertapenem susceptibility. However, recent reports have also shown increasing ertapenem resistance of ESBL isolates. This suggests that ertapenem, with its ease of dosing and improved cost, may be an acceptable alternative to other carbapenems in the treatment of serious infections, but should be used with caution after further susceptibility testing. Clinical trials assessing the use of ertapenem to treat serious infections caused by ESBL isolates are needed.

## Abbreviations

ESBL: Extended spectrum beta-lactamase

Meropenem: MEM

Imipenem: IPM

Ertapenem: ETP

Walter Reed Army Medical Center: WRAMC

## Competing interests

The author(s) declare that they have no competing interests.

## Authors' contributions

All authors have read and approved the final manuscript.

RM participated in the analysis of data and coordinated and drafted the manuscript.

DE helped to coordinate the study, participated in statistical analysis of data, and helped draft the manuscript.

AS, HC, ES, AE participated in designing the study, accumulating ertapenem susceptibility data, and approving the final manuscript.

KM conceived of the study, participated in its design, and helped draft the manuscript.

## References

[B1] Aronson N, Sanders J, Moran K (2006). In Harm's Way: Infections in Deployed American Military Forces. Clin Inf Dis.

[B2] Rupp M, Fey P (2003). Extended Spectrum B-lactamase (ESBL)-Producing Enterobacteriaceae: Considerations for Diagnosis, Prevention, and Drug Treatment. Drugs.

[B3] Colodner R, Raz R (2005). Extended-Spectrum Beta-Lactamases: The End of Cephalosporins?. IMAJ.

[B4] Jacoby G, Munoz-Price L (2005). Mechanisms of Disease: The New (beta)-Lactamases. N Eng J Med.

[B5] Ramphal R, Ambrose P (2006). Extended-Spectrum B-Lactamases and Clinical Outcomes: Current Data. Clin Inf Dis.

[B6] Betriu C, Salso S, Sancez A, Culebras E, Gomez M, Rodriguez-Avial I, Picazo J (2006). Comparative in vitro activity and the inoculum effect of ertapenem against Enterobacteriaceae resistant to extended-spectrum cephalosporins. Int J Antimicrob Agents.

[B7] Livermore D, Oakton K, Carter M, Warner M (2001). Activity of Ertapenem (MK-0826) versus Enterobacteriaceae with Potent B-Lactamases. Antimicrob Agents Chemother.

[B8] Clinical and Laboratory Standards Institute (CLSI) (2006). Performance Standard for Antimicrobial Susceptibility Testing. 16^th ^Informational supplement. CLSI document M100-S16 Wayne, PA.

[B9] (2006). AB BIODISK ETest Ertapenem Package Insert. AB BIODISK, Solna, Sweden.

[B10] Elliott E, Brink A, van Greune J, Els Z, Woodford N, Turton J, Warner M, Livermore DM (2006). In Vivo Development of Ertapenem Resistance in a Patient with Pneumonia Caused by *Klebsiella pneumoniae *with an Extended-Spectrum *B*-lactamase. Clin Inf Dis.

[B11] Jacoby G, Mills D, Chow N (2004). Role of *B*-Lactamases and Porins in Resistance to Ertapenem and Other B-Lactams in *Klebsiella pneumoniae*. Antimicrob Agents Chemother.

[B12] Lartigue M, Poirel L, Poyart C, Reglier-Poupet H, Nordmann P (2007). Ertapenem Resistance of Escherichia coli. Emerging Inf Dis.

[B13] Paterson D, Rossi F, Baquero F, Hsueh P, Woods GL, Satishchandran V, Snyder TA, Harvey CM, Teppler H, DiNubile MJ, Chow JW (2005). In vitro susceptibilities of aerobic and faculatative Gram-negative bacilli isolated from patients with intra-abdominal infections worldwide: the 2003 Study for Monitoring Antimicrobial Resistance Trends (SMART). J Antimicrob Chemother.

